# Age- and cell cycle-related expression patterns of transcription factors and cell cycle regulators in Müller glia

**DOI:** 10.1038/s41598-022-23855-w

**Published:** 2022-11-15

**Authors:** Maki Kato, Norihiro Sudou, Kaori Nomura-Komoike, Tomohiro Iida, Hiroki Fujieda

**Affiliations:** 1 Department of Anatomy and Neurobiology, School of Medicine, Tokyo Women’s Medical University, 8-1 Kawada-Cho, Shinjuku-Ku, Tokyo, 162-8666 Japan; 2 Department of Ophthalmology, School of Medicine, Tokyo Women’s Medical University, Tokyo, Japan; 3 Department of Anatomy, School of Medicine, Toho University, Tokyo, Japan

**Keywords:** Cell biology, Developmental biology, Neuroscience

## Abstract

Mammalian Müller glia express transcription factors and cell cycle regulators essential for the function of retinal progenitors, indicating the latent neurogenic capacity; however, the role of these regulators remains unclear. To gain insights into the role of these regulators in Müller glia, we analyzed expression of transcription factors (Pax6, Vsx2 and Nfia) and cell cycle regulators (cyclin D1 and D3) in rodent Müller glia, focusing on their age- and cell cycle-related expression patterns. Expression of Pax6, Vsx2, Nfia and cyclin D3, but not cyclin D1, increased in Müller glia during development. Photoreceptor injury induced cell cycle-associated increase of Vsx2 and cyclin D1, but not Pax6, Nfia, and cyclin D3. In dissociated cultures, cell cycle-associated increase of Pax6 and Vsx2 was observed in Müller glia from P10 mice but not from P21 mice. Nfia levels were highly correlated with EdU incorporation suggesting their activation during S phase progression. Cyclin D1 and D3 were transiently upregulated in G1 phase but downregulated after S phase entry. Our findings revealed previously unknown links between cell cycle progression and regulator protein expression, which likely affect the cell fate decision of proliferating Müller glia.

## Introduction

Retinal Müller glia have a capacity to regenerate neurons after injury. However, this capacity of Müller glia varies widely across species. In zebrafish, injury induces Müller glia to proliferate and generate neurogenic progenitors that differentiate into neurons to restore retinal structure and function^1,2^. In contrast, mouse Müller glia rarely divide in response to injury^3–5^ and rat Müller glia, although they proliferate after injury, quickly exit the cell cycle and many of the progeny die possibly due to the DNA damage response^5^. The proliferative and neurogenic competence of mammalian Müller glia could be enhanced by mitogen stimulation^6–9^, forced expression of proneural bHLH transcription factors^10–13^, or Hippo pathway inactivation^14,15^. However, reprogramming mammalian Müller glia to highly regenerative progenitors comparable to those of zebrafish remains extremely challenging. Understanding the endogenous molecular mechanisms regulating the injury-induced responses of Müller glia would be crucial to develop strategies to activate the regenerative potential of the mammalian retina.

Müller glia are strikingly similar to retinal progenitor cells (RPC) in gene expression^16,17^. For example, transcription factors Pax6 and Vsx2 are classical RPC markers essential for RPC proliferation^18–20^ and are both expressed in Müller glia^17,21^. The presence of these RPC regulators may indicate the latent capacity of mammalian Müller glia to proliferate and regenerate neurons. Indeed, rodent Müller glia forced to proliferate in dissociated cultures upregulate these transcription factors supporting their role in maintaining a proliferative nature of Müller glia^22–24^. Also, injury induces upregulation of Pax6 and Vsx2 in Müller glia, which has been considered a hallmark of Müller glia dedifferentiation/reprograming toward the progenitor state^4,6,8,25,26^. However, a recent single-cell transcriptome analysis has indicated that both Pax6 and Vsx2 belong to a group of transcription factors promoting quiescence of Müller glia after injury^27^. This surprising finding contradicts the conventional view that Pax6 and Vsx2 promote reprogramming of Müller glia. However, the study by Hoang et al.^27^ utilized in vivo mouse models of retinal injury where few, if any, Müller glia are capable of proliferation, and thus the role of these RPC transcription factors in proliferating Müller glia remains unclear. Indeed, in zebrafish, Pax6 is required for the proliferation of Müller glia-derived progenitors, but not for the initial Müller glia division after injury^28^. Thus, these RPC transcription factors may function in a context-dependent manner and change their roles as Müller glia traverse different phases of their injury-induced responses. The NFI family of transcription factors (Nfia, Nfib, and Nfix) are also expressed in RPC and Müller glia^21,29^ and have been shown to induce cell cycle exit of RPC and specification of Müller glia and bipolar cells^30^. These factors, like Pax6 and Vsx2, have been shown to promote quiescence of Müller glia after injury^27^. However, NFI factors are highly expressed in Müller glia-derived proliferating progenitors in the chick retina, arguing against the role of these factors in the induction of cell cycle exit^31^. Thus, again, the role of these factors may change once Müller glia reenter the cell cycle and dedifferentiate into progenitor-like cells.

Expression of D-type cyclins is induced by extracellular mitogens and promotes G1 phase progression by activating cyclin-dependent kinases (CDK) 4/6^32^. Growing evidence has also indicated that both D1 and D3 cyclins can act as transcriptional regulators controlling developmental gene expression^33–37^. Cyclin D1 is the predominant D-type cyclin in RPC and essential for RPC proliferation^38,39^. Cyclin D1 is rapidly downregulated upon cell cycle exit of RPC, but is retained, albeit at lower levels, in postmitotic Müller glia^5,40^. In contrast, cyclin D3 is absent in RPC, but is upregulated in Müller glia as they differentiate^40^. The precise roles of these cell cycle regulators in Müller glia remain unclear, but their expression patterns after injury have been documented extensively. While cyclin D1 has been consistently shown to increase in Müller glia after injury^3–5,14,15,41,42^, there is a discrepancy in the literature regarding cyclin D3 expression; one study reported its downregulation after injury^43^ while most others claim its upregulation^3,4,15,41,42^. Like transcription factors described above, expression of cell cycle regulators has been studied mostly in mice, whose Müller glia have extremely limited proliferative potential in vivo. Information about expression of these cell cycle regulators during Müller glia proliferation may help understand their roles and mechanisms restricting the proliferative potential of mammalian Müller glia.

To gain insights into the context-dependent mechanisms regulating the reprogramming responses of Müller glia, we analyzed protein expression of RPC transcription factors and cell cycle regulators in rodent Müller glia in vivo and in vitro, focusing on their age- and cell cycle-related expression changes. Our findings revealed previously unknown links between cell cycle progression and regulator protein expression, which likely affect the cell fate decision of proliferating Müller glia.

## Results

### Expression of RPC transcription factors and cell cycle regulators in Müller glia during development

**Figure 1:**
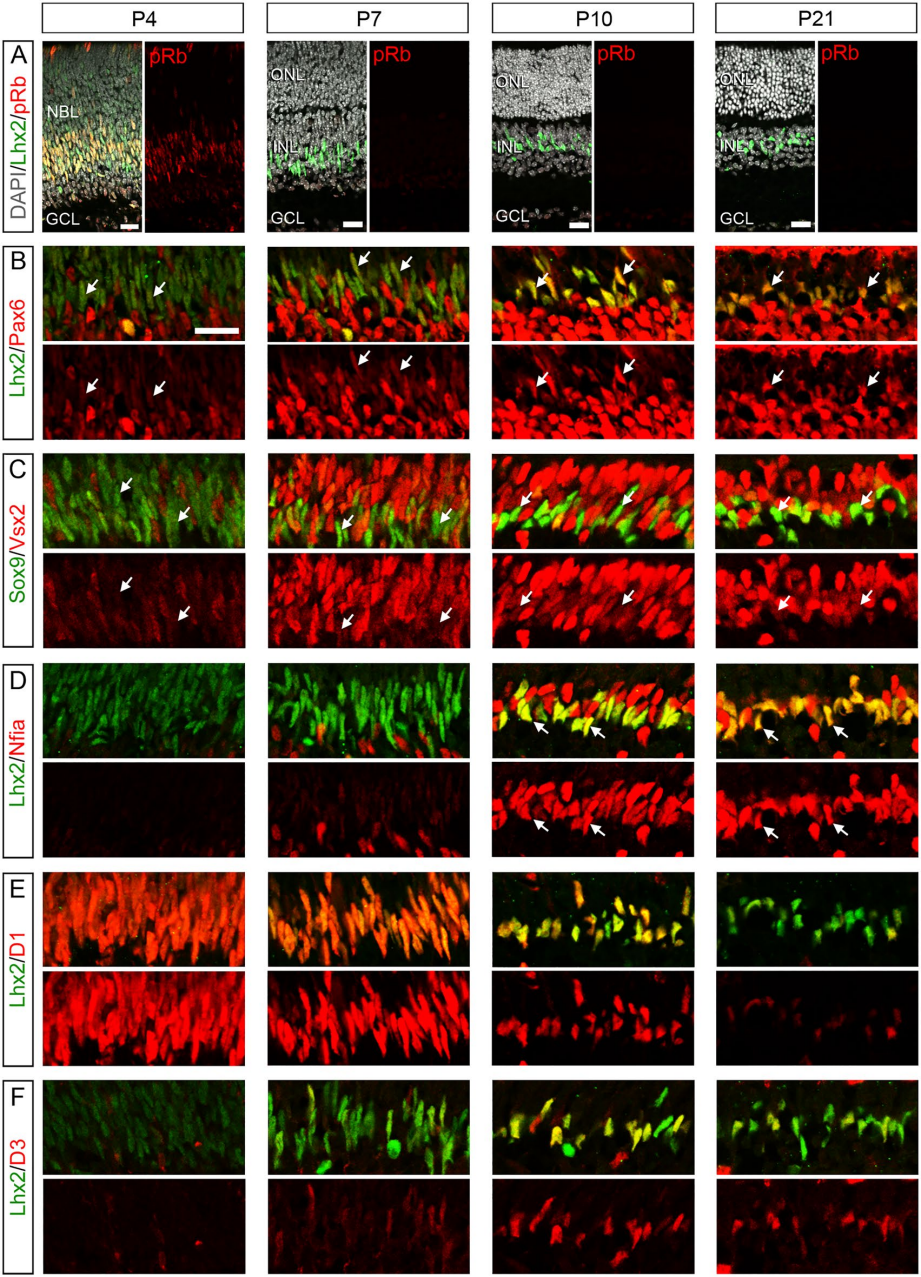
Immunofluorescence for RPC transcription factors and cell cycle regulators in Müller glia during development. (**A**) Lhx2+ cells at P4 are mostly phospho-pRb (pRb) + RPC while those after P7 are pRb− Müller glia. (**B**) Pax6 immunoreactivity in Lhx2 + RPC and Müller glia (arrows). (**C**) Vsx2 immunoreactivity in Sox9 + RPC and Müller glia (arrows). (**D**) Nfia immunoreactivity in Lhx2 + RPC and Müller glia (arrows). (**E**) Cyclin D1 immunoreactivity in Lhx2 + RPC and Müller glia. (**F**) Cyclin D3 immunoreactivity in Lhx2 + RPC and Müller glia. *NBL* neuroblastic layer, *GCL* ganglion cell layer, *ONL* outer nuclear layer, *INL* inner nuclear layer. Scale bar = 20 µm.

We first analyzed expression of RPC transcription factors (Pax6, Vsx2 and Nfia) and cell cycle regulators (cyclin D1 and D3) in RPC and Müller glia during postnatal mouse development by immunofluorescence. As all of the transcription factors examined are expressed in subsets of postmitotic neurons as well as RPC/Müller glia, RPC/Müller glia-specific markers Sox9 or Lhx2 were included in the staining. Phospho-pRb, a cell cycle marker, served to differentiate proliferating RPC from postmitotic Müller glia. At P4, most Lhx2+ cells were phospho-pRb+, indicating the RPC identity (Fig. 1A). Consistent with the previous report^44^, RPC proliferation was no longer found by P7 in the central retina and P10 in the far periphery. Thus, all Lhx2/Sox9+ cells in the central retina (except a small number of Lhx2+ amacrine cells) were considered as Müller glia after P7 (Fig. 1A). Pax6 immunoreactivity was relatively weak in RPC but drastically increased in differentiating Müller glia by P10 (Fig. 1B). Vsx2 immunoreactivity was also weak in RPC and increased during Müller glia differentiation, but this increase was not so drastic as Pax6 (Fig. 1C). Nfia was barely detectable in RPC but increased dramatically during Müller glia differentiation by P10, similar to the changes in Pax6 (Fig. 1D). As opposed to the above transcription factors, cyclin D1 was abundantly expressed in RPC and decreased drastically during Müller glia differentiation (Fig. 1E). In contrast, cyclin D3 was not detectable in RPC, but increased by P10 as Müller glia mature, showing a similar temporal pattern to Pax6 or Nfia (Fig. 1F). Together, these data show that the expression of Pax6, Vsx2 and Nfia as well as cyclin D3 increased as Müller glia differentiate while cyclin D1 expression declined with Müller glia maturation.

### Expression of RPC transcription factors and cell cycle regulators in Müller glia after photoreceptor injury

**Figure 2:**
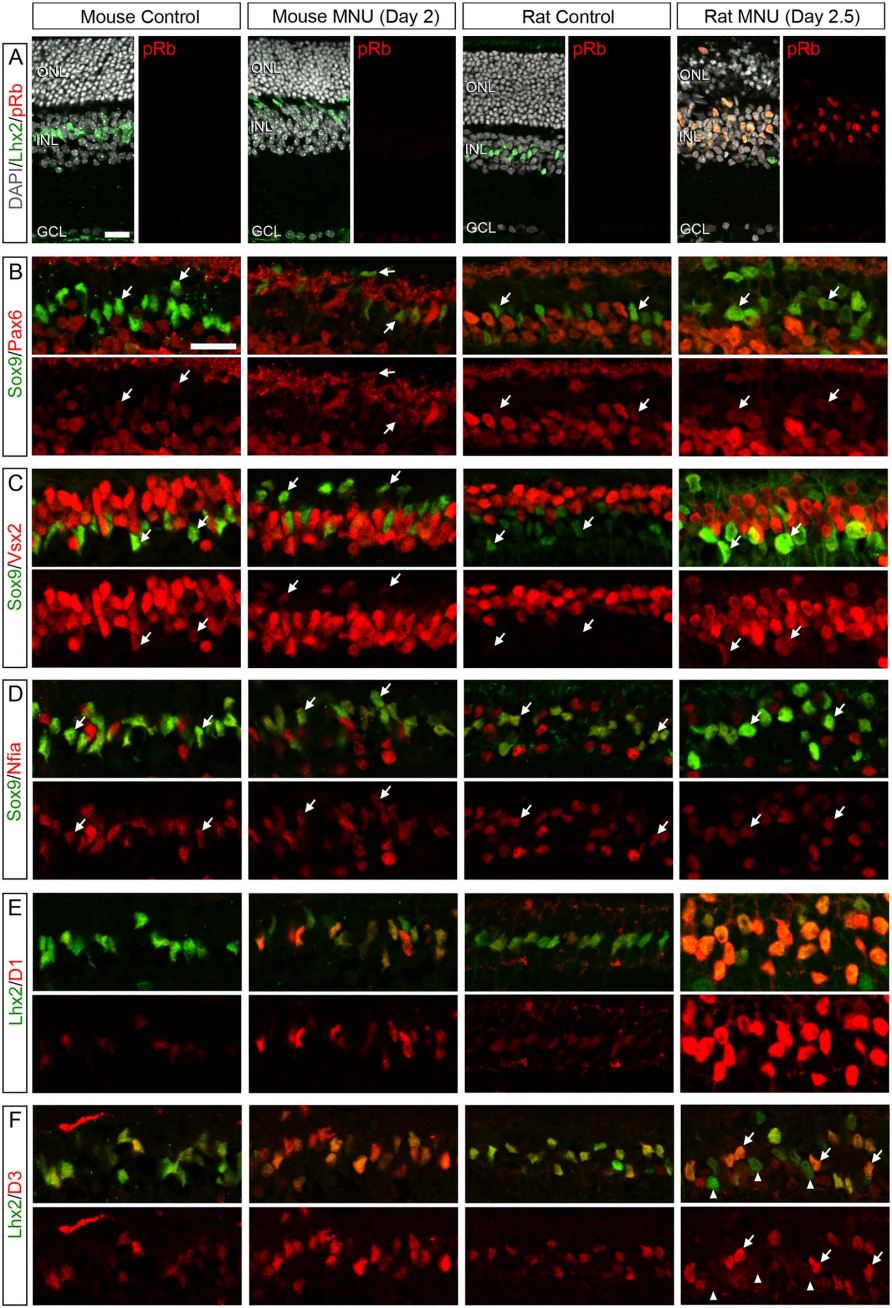
Immunofluorescence for RPC transcription factors and cell cycle regulators in Müller glia after MNU-induced injury in mice and rats. (**A**) Phospho-pRb (pRb) immunoreactivity in Lhx2 + Müller glia. (**B**) Pax6 immunoreactivity in Sox9 + Müller glia (arrows). (**C**) Vsx2 immunoreactivity in Sox9 + Müller glia (arrows). (**D**) Nfia immunoreactivity in Sox9 + Müller glia (arrows). (**E**) Cyclin D1 immunoreactivity in Lhx2 + Müller glia (arrows). (**F**) Cyclin D3 immunoreactivity in Lhx2 + Müller glia. Some Müller glia were intensely labeled for cyclin D3 (arrows) while others were negative or only weakly positive (arrowheads) in the rat retina after injury. *ONL* outer nuclear layer, *INL *inner nuclear layer, *GCL* ganglion cell layer. Scale bar = 20 µm.

We next examined expression changes of transcription factors and cell cycle regulators in the mouse and rat retinas after *N*-methyl-*N*-nitrosourea (MNU)-induced photoreceptor injury. We previously reported that Müller glia reenter the cell cycle after MNU-induced photoreceptor injury in rats, but not in mice^5^. We took advantage of these rodent models to assess regulator expression changes in Müller glia during their proliferative (rats) and non-proliferative (mice) responses after injury. In agreement with the previous report^5^, the structure of the outer nuclear layer (ONL) in mice was relatively well preserved at day 2 after MNU treatment while that in rats was severely disrupted by day 2.5 possibly due to the prompt removal of dead photoreceptors (Fig. 2A). Müller glia nuclei were displaced toward the outer half of the inner nuclear layer (INL) or the ONL after injury in both mice and rats (Fig. 2A). Immunofluorescence for phospho-pRb confirmed lack of proliferation in the mouse retinas while, in rats, virtually all Müller glia became phospho-pRb + at day 2.5 after MNU treatment (Fig. 2A), consistent with the previous report that most Müller glia enter S phase at this timing^5^. In the mature retina, Pax6 is highly expressed in neurons such as ganglion, amacrine, and horizontal cells. In both mice and rats, Pax6 immunoreactivity in Müller glia was relatively weak compared to neurons and its expression changes after injury was not visually apparent (Fig. 2B). Vsx2 is expressed in bipolar neurons and less abundantly in Müller glia. In mice, Vsx2 immunoreactivity in Müller glia did not alter significantly after injury (Fig. 2C). In rats, by contrast, Vsx2 immunoreactivity increased in most Müller glia by day 2.5 after injury (Fig. 2C). Nfia has been localized to Müller glia and some amacrine and bipolar neurons^29^. Nfia immunoreactivity in Müller glia did not change significantly after injury in both mice and rats (Fig. 2D). Cyclin D1 immunoreactivity in Müller glia was increased after injury in both mice and rats, but this increase was much more dramatic in rats (Fig. 2E). Cyclin D3 immunoreactivity was also increased in most Müller glia after injury in mice (Fig. 2F). In rats, by contrast, the changes in cyclin D3 levels were highly heterogeneous among cells; cyclin D3 was prominently increased in some Müller glia while decreasing to undetectable levels in others (Fig. 2F).

### Expression of RPC transcription factors and cell cycle regulators in dissociated cultures of Müller glia

**Figure 3:**
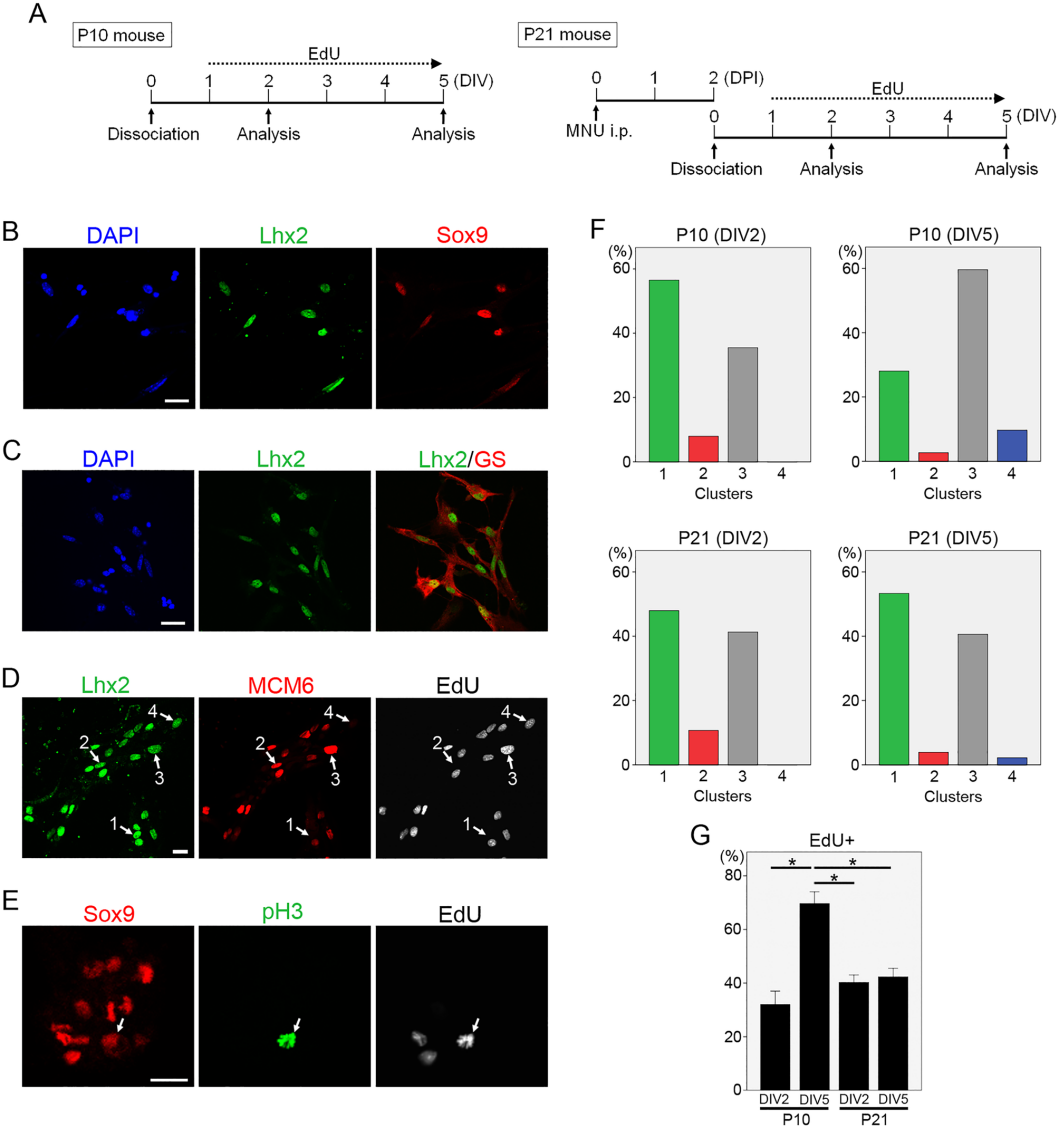
Dissociated cultures of mouse Müller glia. (**A**) Diagrams showing experimental design. P10 and P21 mouse retinas were dissociated and cultured in the continuous presence of EdU. P21 retinas were dissociated 2 days after MNU treatment. Cell were fixed and analyzed by immunofluorescence at 2 days in vitro (DIV2) and DIV5. DPI, days post-injection. (**B**) Colocalization of Müller glia markers Lhx2 and Sox9. Scale bar = 20 µm. (**C**) Colocalization of Müller glia markers Lhx2 and glutamine synthetase (GS). Scale bar = 20 µm. (**D**) Lhx2 + Müller glia were classified into four clusters based on MCM6 and EdU labeling (arrows with numbers). Scale bar = 20 µm. (**E**) Phospho-histone H3 (pH3) + Müller glia in M phase (arrows). Scale bar = 20 µm. (**F**) Histograms showing the proportions of each cluster. Totally 3796 cells (DIV2) and 5554 cells (DIV5) from P10 retinas and 5250 cells (DIV2) and 3864 cells (DIV5) from P21 retinas were analyzed. (**G**) Proportions of EdU + Müller glia. Each bar represents the mean ± SEM, P* < 0.05.

Because mouse Müller glia rarely proliferate in vivo even after injury, we attempted to induce cell cycle reentry of mouse Müller glia by dissociation cultures. The retinas from P10 and P21 mice were dissociated and cultured for two (DIV2) to five days (DIV5) in the presence of EdU (Fig. 3A). Müller glia dissociated from P10 mice grew well, but we failed to establish Müller glia cultures from P21 mouse retinas, in agreement with the previous report^45^. However, we could obtain Müller glia cultures from P21 mouse retinas by prior induction of photoreceptor injury by MNU. When dissociated cultures were double-stained for Lhx2 and Sox9, virtually all Lhx2+ cell nuclei were Sox9+ and virtually all Sox9+ cell nuclei were Lhx2+ (Fig. 3B). We also confirmed that Lhx2 was colocalized with cytoplasmic Müller glia marker glutamine synthetase (Fig. 3C). Thus, we concluded that Müller glia/RPC markers Lhx2 and Sox9 specifically labelled the Müller glia nuclei in dissociated cultures from mouse retinas.

We next sought to characterize cell cycle progression of dissociated Müller glia by assessing EdU incorporation and immunofluorescence for a pan-cell cycle marker MCM6^5,46^ (Fig. 3D). We also examined M phase entry by phospho-histone H3 (pH3) staining (Fig. 3E). As cells were cultured in the continuous presence of EdU (Fig. 3A), the presence of EdU labeling indicates that the cell entered S phase at least once. EdU intensity reflects the extent of DNA replication during S phase; cells in late S phase are expected to exhibit higher EdU intensity than those in early S phase, and those progressing through the second round of DNA replication accumulate more EdU than the first round^47,48^. The absence of EdU labeling denotes that the cell is quiescent (G0) or in G1 phase of the first cell cycle. Based on EdU and MCM6 labeling, Müller glia in dissociated cultures were grouped into four clusters: EdU−/MCM6− (cluster 1), EdU-MCM6+ (cluster 2), EdU+/MCM6+ (cluster 3), and EdU+/MCM6− (cluster 4). Most EdU− cells were MCM6− (cluster 1), but we noted a small EdU−/MCM6 + population (cluster 2) (Fig. 3D,F). As MCM6 has been reported to increase in late G1^49^, the cluster 2 was considered to include cells in late G1 of the first cell cycle. When assessed at DIV2, all EdU+ Müller glia were MCM6+ (cluster 3) while a small EdU+/MCM6− population (cluster 4) was found at DIV5, most likely representing cells that exited the cell cycle after S phase entry (Fig. 3D,F). When Müller glia from P10 retinas were analyzed, 35% at DIV2 and 70% at DIV5 were EdU+, indicating a progression of S phase entry during the culture period of five days (Fig. 3G). In contrast, the cell cycle distributions of Müller glia from P21 retinas were similar between DIV2 and DIV5 except the appearance of the cluster 4 at DIV5; approximately half of Müller glia remained in G0/early G1 (cluster 1) at both DIV2 and DIV5 (Fig. 3F,G). The proportions of pH3 + Müller glia were not significantly different between P10 and P21 samples when analyzed at DIV2 [P10: 0.29% ± 0.19% vs P21: 0.37% ± 0.04% (mean ± s.e.m.)]. At DIV5, however, pH3 + Müller glia were virtually absent in both P10 and P21 samples.

**Figure 4:**
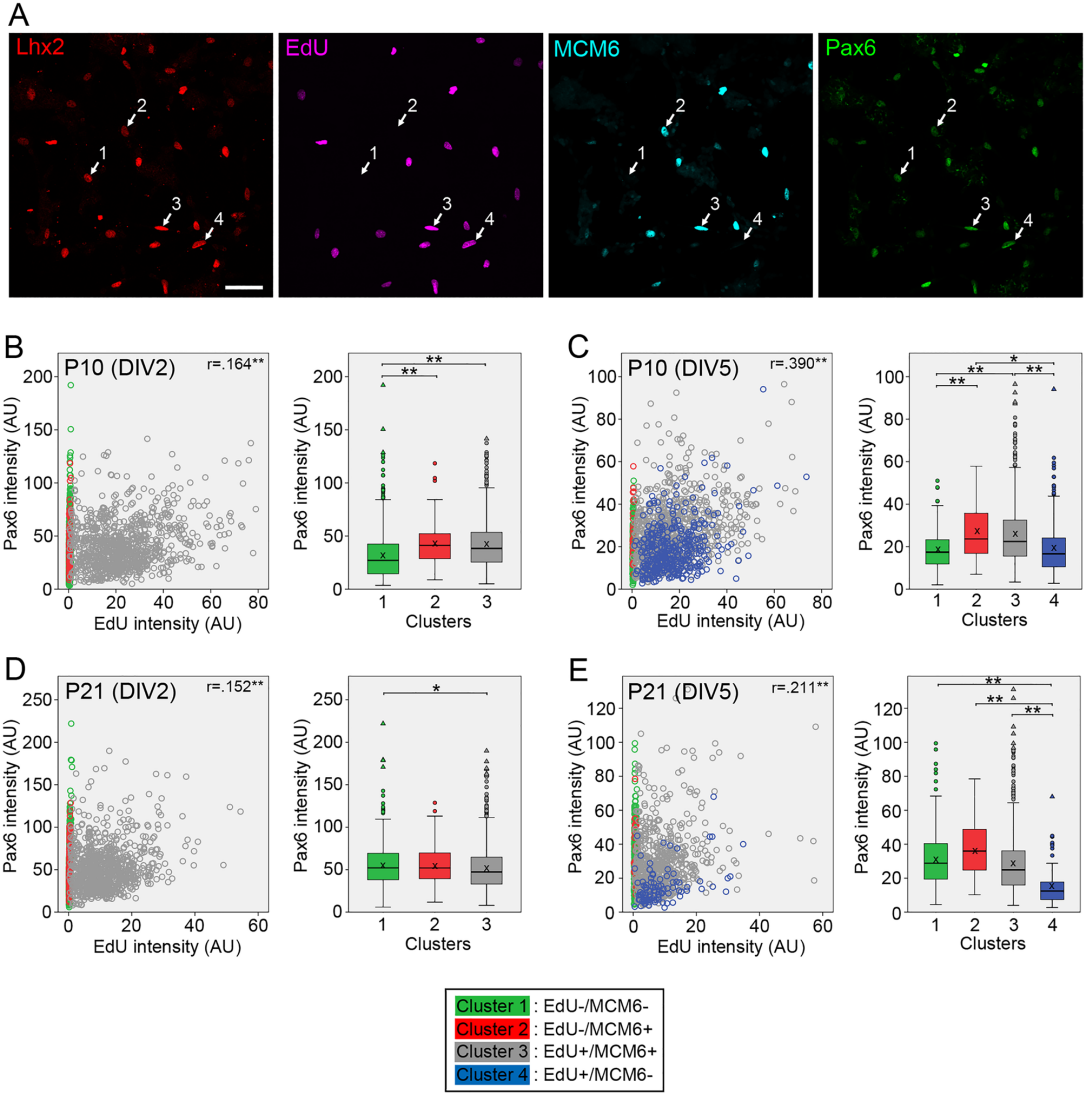
Quantitative analyses of Pax6 expression in Müller glia dissociated from P10 and P21 mouse retinas. Intensities of EdU, MCM6 and Pax6 labeling in Lhx2 + Müller glia were quantitated by image analysis. Pax6 levels in Müller glia classified into four clusters are shown as scatter plots and box plots. (**A**) Confocal images of quadruple labeling for Lhx2, EdU, MCM6 and Pax6 in Müller glia from P10 retina at DIV5. The numbers represent the clusters. Scale bar = 50 µm. (**B**) Plots of 2138 Müller glia from P10 retina at DIV2. (**C**) Plots of 1700 Müller glia from P10 retina at DIV5. (**D**) Plots of 1895 Müller glia from P21 retina at DIV2. (**E**) Plots of 1118 Müller glia from P21 retina at DIV5. *AU* arbitrary units. *r*, Spearman’s correlation coefficients between Pax6 and EdU intensities in the cluster 3. X in box plots indicates the mean. *P < 0.05, **P < 0.01.

We next analyzed expression changes of RPC regulators during cell cycle progression of dissociated Müller glia. Quadruple staining for a Müller glia marker (Lhx2 or Sox9), MCM6, EdU, and a regulator protein (Pax6, Vsx2, Nfia, cyclin D1 and cyclin D3) was conducted, and intensities of fluorescent markers were quantitated and compared between the four clusters of Müller glia defined above. We first conducted Pax6 immunofluorescence together with cell cycle markers (Fig. 4A). When P10 samples were analyzed at DIV2, Pax6 levels in the cluster 2 and 3 were significantly higher than those in the cluster 1, indicating higher expression during cell cycle progression compared to quiescence (Fig. 4B). A similar increase of Pax6 levels in the cluster 2/3 was also observed at DIV5, and Pax6 levels in the cluster 4 were significantly lower compared to the cluster 2/3, indicating a decrease in Pax6 levels during cell cycle exit (Fig. 4C). In contrast to P10 samples, Müller glia in P21 samples showed no increase in Pax6 levels in the cluster 2/3 at both DIV2 and DIV5 (Fig. 4D,E). At DIV5, Pax6 levels in the cluster 4 were significantly lower than those in the other clusters (Fig. 4E). The correlations between Pax6 and EdU intensities in the cluster 3 were weak or negligible regardless of age and culture period (Fig. 4B–E).

**Figure 5:**
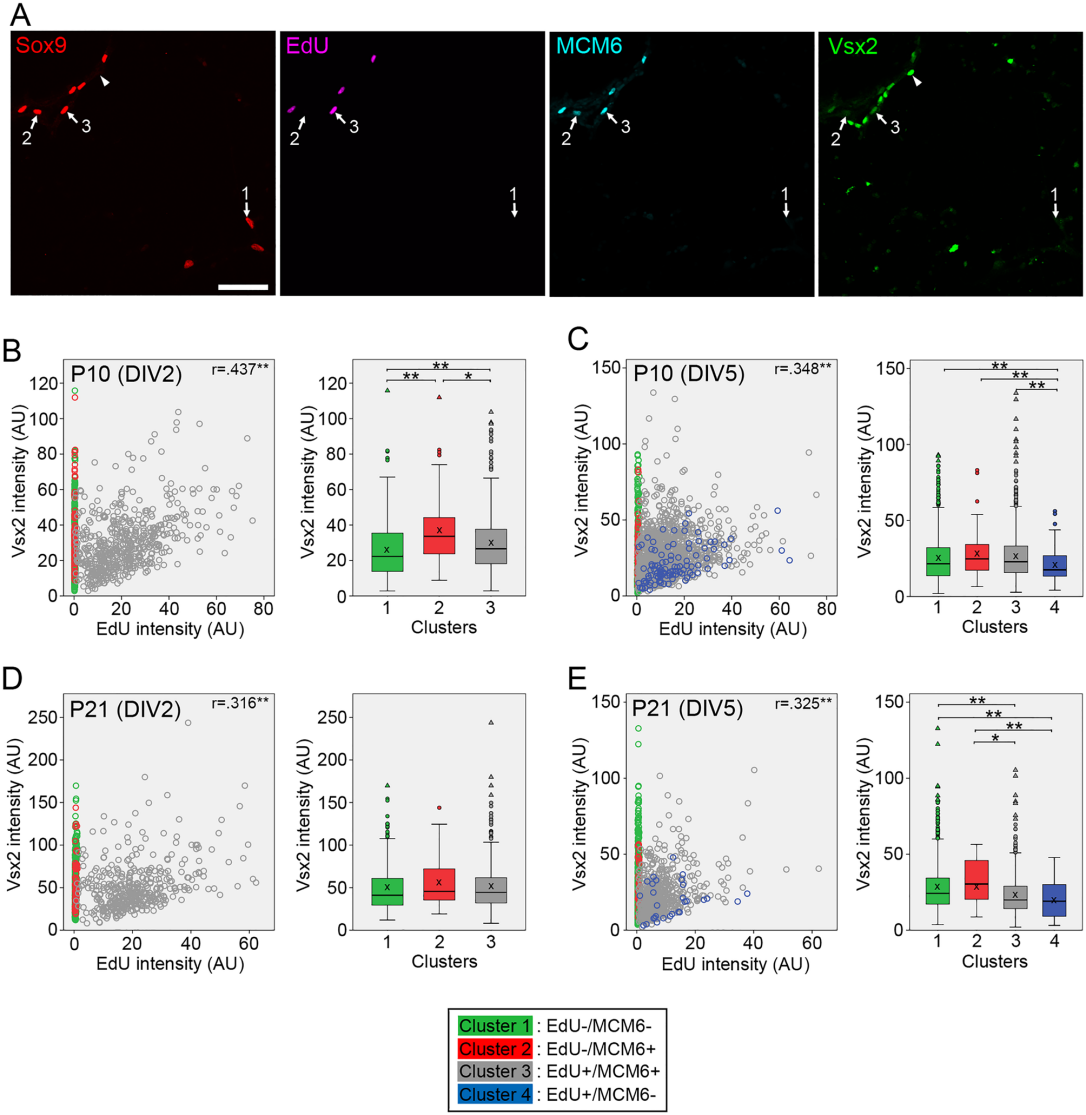
Quantitative analyses of Vsx2 expression in Müller glia dissociated from P10 and P21 mouse retinas. Intensities of EdU, MCM6 and Vsx2 labeling in Sox9 + Müller glia are quantitated by image analysis. Vsx2 levels in Müller glia classified into four clusters are shown as scatter plots and box plots. (**A**) Confocal images of quadruple labeling for Sox9, EdU, MCM6 and Vsx2 in Müller glia from P10 retina at DIV2. The numbers represent the clusters. Arrowheads denote Vsx2+/Sox9− bipolar cells. Scale bar = 50 µm. (**B**) Plots of 1061 Müller glia from P10 retina at DIV2. (**C**) Plots of 2305 Müller glia from P10 retina at DIV5. (**D**) Plots of 916 Müller glia from P21 retina at DIV2. E. Plots of 1354 Müller glia from P21 retina at DIV5. *AU* arbitrary units. *r*, Spearman’s correlation coefficients between Vsx2 and EdU intensities in the cluster 3. X in box plots indicates the mean. *P < 0.05, **P < 0.01.

We next analyzed Vsx2 expression. Many Vsx2+/Sox9− cells were found in both P10 and P21 samples, which were most likely surviving bipolar neurons (Fig. 5A). When P10 samples were analyzed at DIV2, Vsx2 levels in the cluster 2 and 3 were significantly higher than those in the cluster 1 (Fig. 5B), showing a pattern similar to Pax6 levels. The increase in the cluster 2/3 compared to the cluster 1 was no more evident at DIV5 although a decrease in Vsx2 levels associated with cell cycle exit (cluster 4) was significant (Fig. 5C). In contrast to P10 samples, there were no significant differences in Vsx2 levels between the clusters of P21 samples at DIV2 (Fig. 5D). At DIV5, Vsx2 levels in both cluster 3 and 4 were significantly lower than those in the cluster 1 and 2 (Fig. 5E). The correlations between EdU and Vsx2 intensities in the cluster 3 were weak regardless of age and culture period (Fig. 5B–E).

**Figure 6:**
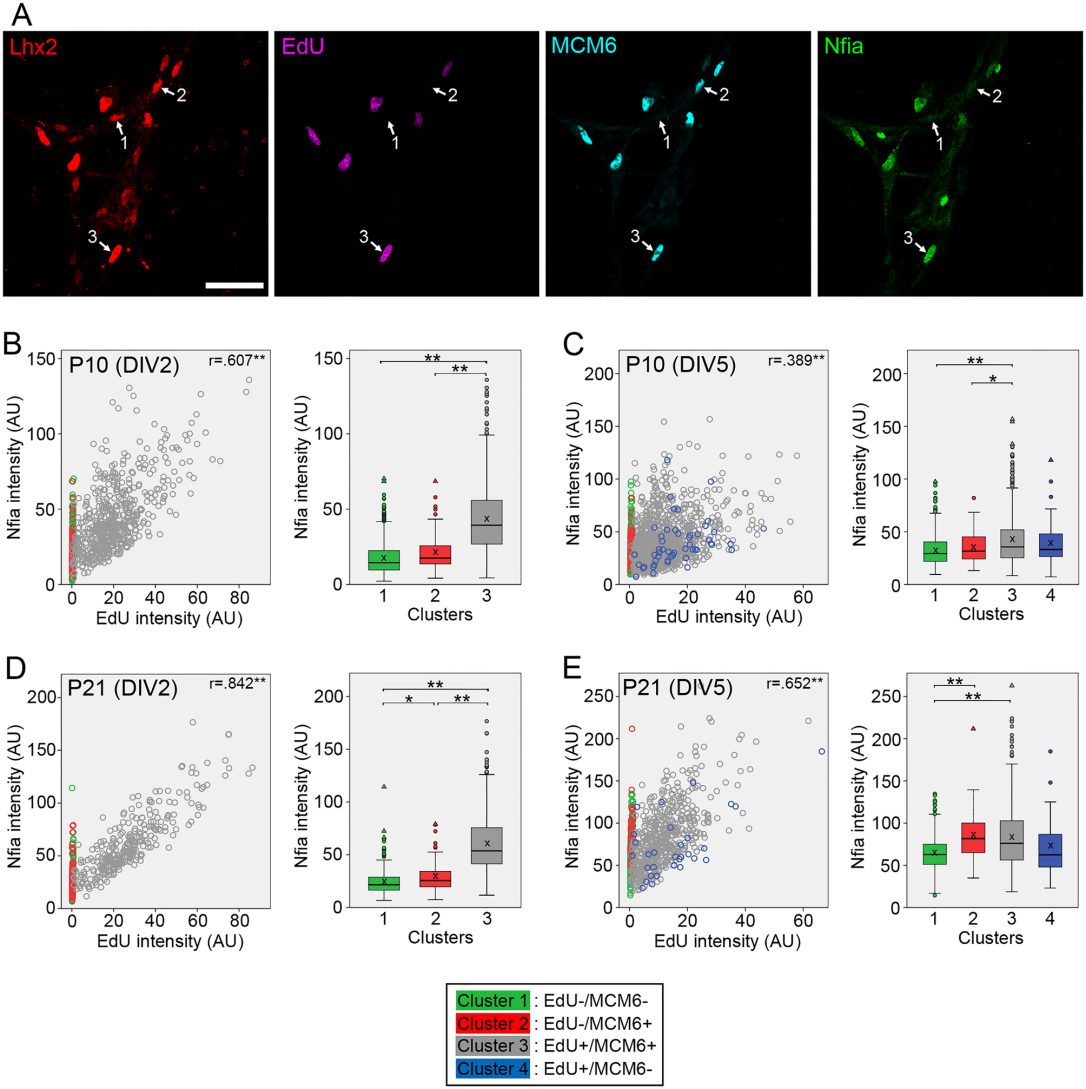
Quantitative analyses of Nfia expression in Müller glia dissociated from P10 and P21 mouse retinas. Intensities of EdU, MCM6 and Nfia labeling in Lhx2 + Müller glia were quantitated by image analysis and Nfia levels in Müller glia classified into four clusters are shown as scatter plots and box plots. (**A**) Confocal images of quadruple labeling for Lhx2, EdU, MCM6 and Nfia in Müller glia from P21 retina at DIV2. The numbers represent the clusters. Scale bar = 50 µm. (**B**) Plots of 1260 Müller glia from P10 retina at DIV2. (**C**) Plots of 1549 Müller glia from P10 retina at DIV5. (**D**) Plots of 671 Müller glia from P21 retina at DIV2. (**E**) Plots of 1198 Müller glia from P21 retina at DIV5. *AU* arbitrary units. *r*, Spearman’s correlation coefficients between Nfia and EdU intensities in the cluster 3. X in box plots indicates the mean. *P < 0.05, **P < 0.01.

Nfia expression in dissociated Müller glia was unique in its upregulation in the cluster 3 (Fig. 6A–E). When examined at DIV2, Nfia levels in the cluster 3 were significantly higher than those in the other clusters in both P10 and P21 samples (Fig. 6B,D). Moreover, the correlations between Nfia and EdU levels in the cluster 3 were moderate (r = 0.607) for P10 samples and strong (r = 0.842) for P21 samples (Fig. 6B,D), indicating Nfia upregulation during S phase progression. A moderate correlation between Nfia and EdU levels was still found for P21 samples examined at DIV5 (Fig. 6E).

**Figure 7:**
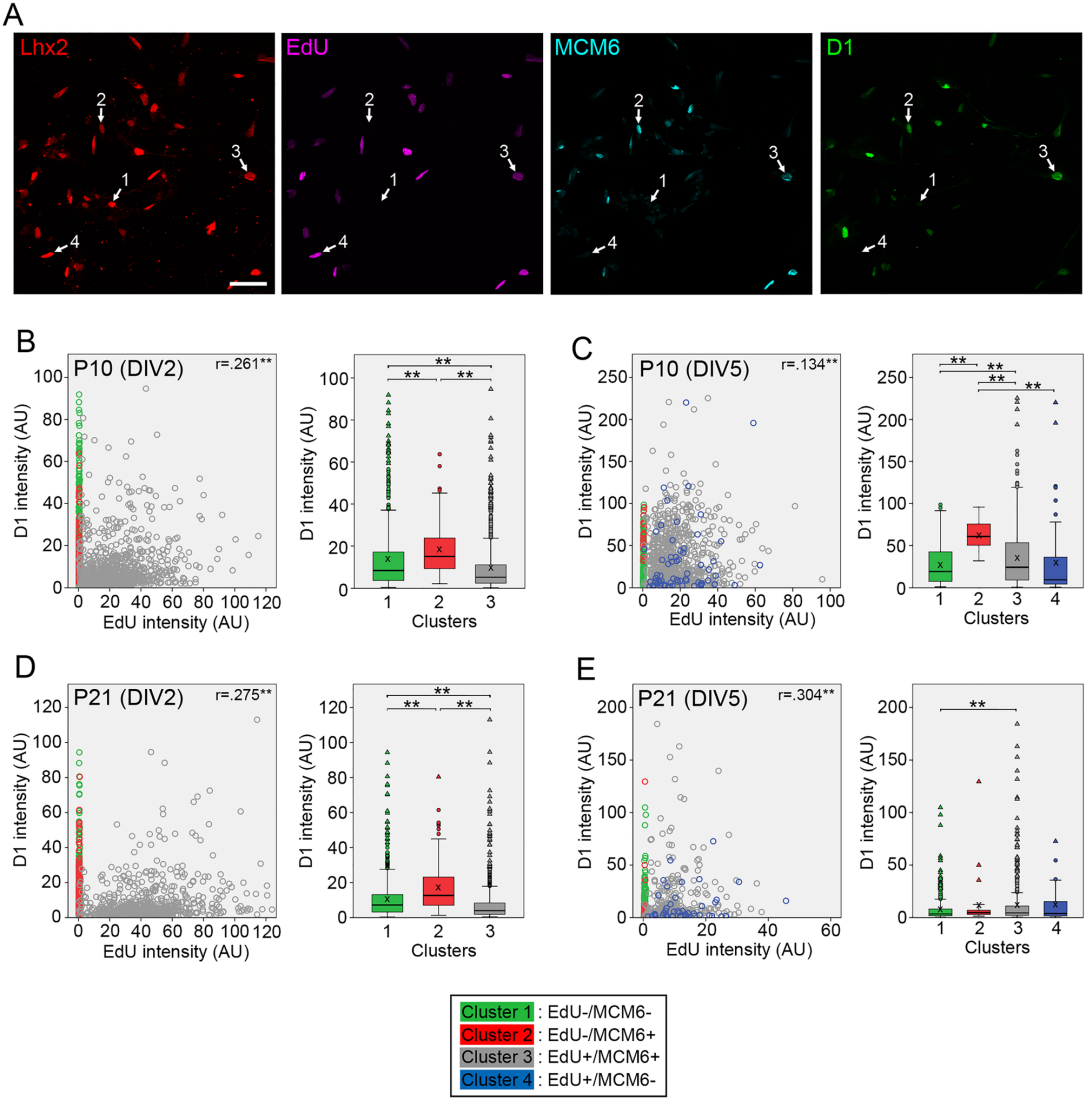
Quantitative analyses of cyclin D1 expression in Müller glia dissociated from P10 and P21 mouse retinas. Intensities of EdU, MCM6 and cyclin D1 labeling in Lhx2 + Müller glia were quantitated by image analysis and cyclin D1 levels in Müller glia classified into four clusters are shown as scatter plots and box plots. (**A**) Confocal images of quadruple labeling for Lhx2, EdU, MCM6 and cyclin D1 in Müller glia from P10 retina at DIV5. The numbers represent the clusters. Scale bar = 50 µm. (**B**) Plots of 1745 Müller glia from P10 retina at DIV2. (**C**) Plots of 1021 Müller glia from P10 retina at DIV5. (**D**) Plots of 1867 Müller glia from P21 retina at DIV2. (**E**) Plots of 921 Müller glia from P21 retina at DIV5. *AU* arbitrary units. *r*, Spearman’s correlation coefficients between cyclin D1 and EdU intensities in the cluster 3. X in box plots indicates the mean. *P < 0.05, **P < 0.01.

**Figure 8:**
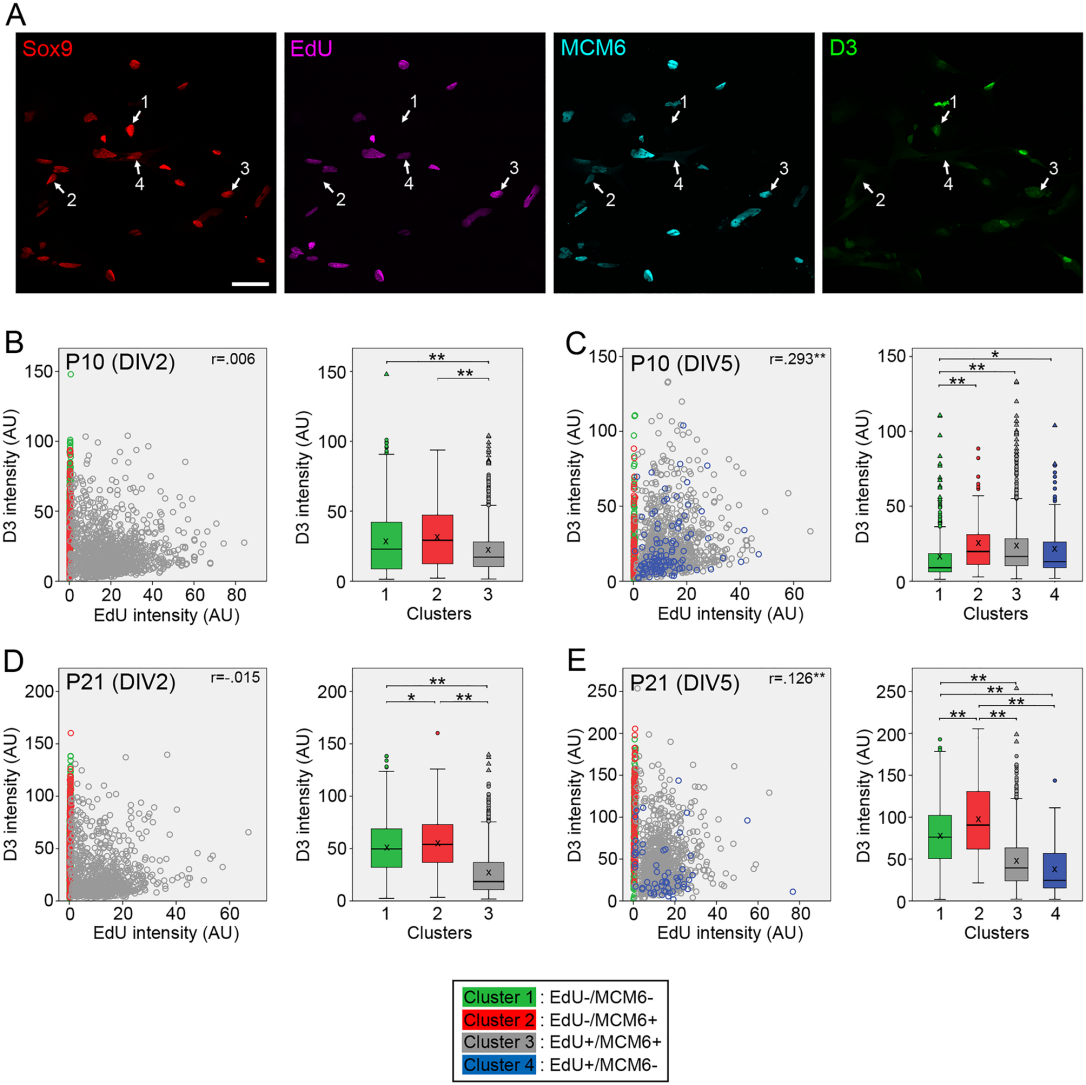
Quantitative analyses of cyclin D3 expression in Müller glia dissociated from P10 and P21 mouse retinas. Intensities of EdU, MCM6 and cyclin D3 labeling in Sox9 + Müller glia were quantitated by image analysis and cyclin D3 levels in Müller glia classified into four clusters are shown as scatter plots and box plots. (**A**) Confocal images of quadruple labeling for Sox9, EdU, MCM6 and cyclin D3 in Müller glia from P10 retina at DIV5. The numbers represent the clusters. Scale bar = 50 µm. (**B**) Plots of 2432 Müller glia from P10 retina at DIV2. (**C**) Plots of 1607 Müller glia from P10 retina at DIV5. (**D**) Plots of 1918 Müller glia from P21 retina at DIV2. (**E**) Plots of 1646 Müller glia from P21 retina at DIV5. *AU* arbitrary units. *r*, Spearman’s correlation coefficients between cyclin D3 and EdU intensities in the cluster 3. X in box plots indicates the mean. *P < 0.05, **P < 0.01.

Finally, we examined expression of cyclin D1 and D3 in dissociated Müller glia. Consistent with the role of D-type cyclins in G1 phase, cyclin D1 levels were significantly upregulated in the cluster 2 compared to the cluster 1, and subsequently downregulated in the cluster 3 (Fig. 7A–D), except this upregulation in the cluster 2 was not observed in P21 samples at DIV5 (Fig. 7E). Correlations between cyclin D1 and EdU levels were very weak (Fig. 7B–E). Compared to cyclin D1, upregulation of cyclin D3 in the cluster 2 was slight or not significant; however, similar to cyclin D1, cyclin D3 levels were significantly downregulated in the cluster 3, which was more notable in the P21 samples (Fig. 8A–E). Correlations between cyclin D3 and EdU levels were not significant or very weak (Fig. 8B–E).

## Discussion

**Figure 9:**
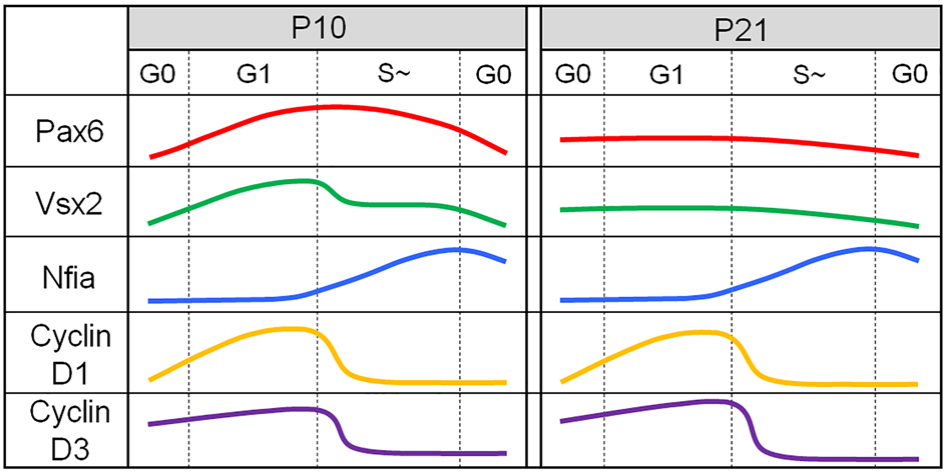
A schematic diagram summarizing expression patterns of RPC transcription factors and cell cycle regulators in dissociated Müller glia during cell cycle progression.

There are a number of previous reports describing expression of RPC regulators in Müller glia after injury^3,8,27^, in retinal explants^4,26^, or in dissociated cultures^22–24,45^. However, none of these studies addressed the association of regulator protein expression with cell cycle progression. To our knowledge, this study is the first to analyze cell cycle-related expression patterns of RPC regulators in Müller glia (Fig. 9). Perhaps the most surprising result of our study was moderate to strong correlations between Nfia expression and EdU incorporation in proliferating Müller glia. This suggests that Nfia expression may be activated in a manner dependent on DNA replication during S phase. DNA replication has been suggested to provide an opportunity for transcriptional reprogramming^50,51^. For example, HoxB genes are activated in S phase and require DNA replication for their expression^52^. Requirement of S phase for lineage-specific gene activation has also been reported in hematopoietic progenitors^53^. Although the mechanism regulating Nfia expression in S phase remains to be studied, our findings reveal a previously unidentified role of S phase in the control of lineage-specific transcription factor expression in Müller glia, which likely affects their regenerative potential after injury. It would be interesting to study whether this S phase-linked upregulation is unique to Nfia or common to other gliogenic factors including Nfib and Nfix.

Pax6 and Vsx2 are well-investigated transcription factors required for RPC proliferation^18–20^. These factors have been reported to increase in Müller glia after injury and considered as Müller glia reprogramming factors^4,8,26^. However, the recent study^27^ suggested that both Pax6 and Vsx2 promote quiescence rather than reprogramming of Müller glia after injury, and the functional significance of these “reprogramming factors” in Müller glia remains unclear. In theory, if a transcription factor acts to reprogram Müller glia toward the progenitor state, it should be highly expressed in RPC and downregulated during Müller glia differentiation. On the contrary, we found that the expression of both Pax6 and Vsx2 was increased as Müller glia mature, supporting a role for these factors in Müller glia differentiation rather than reprogramming. However, when Müller glia were forced to proliferate by injury in vivo (rats) or dissociation culture (P10 mice), these transcription factors were upregulated during cell cycle progression. Considering that both Pax6 and Vsx2 are upregulated during G1/S transition (cluster 2 and 3) and downregulated after cell cycle exit (cluster 4) in dissociated cultures of P10 retinas, these factors are unlikely to promote cell cycle exit of Müller glia. Our data demonstrate close associations between expression of these transcription factors and cell cycle progression of Müller glia, but do not reveal their causal relationships. Nevertheless, several lines of evidence argue against the possibility that upregulation of Pax6 and Vsx2 is essential for the initial cell cycle reentry of Müller glia. First, our data showed that Müller glia dissociated from the P21 mouse retinas proliferated without apparent upregulation of these transcription factors. Second, a previous study reported that, when P8 mouse retinas were explant-cultured in the presence of EGF, approximately 90% of Müller glia proliferated while only 30% expressed Pax6 or Vsx2^26^. Third, in zebrafish, Pax6 is required for the expansion of Müller glia-derived progenitors, but is dispensable for the initial division of Müller glia after injury^28^. Thus, we favor the possibility that cell cycle progression induces upregulation of these transcription factors, which may promote reprogramming of Müller glia after their cell cycle reentry. Age-dependent decline of the neurogenic potential of mouse Müller glia has been reported^26,45^. Lack of cell cycle-associated upregulation of neurogenic transcription factors like Pax6 and Vsx2 in mature Müller glia may account, at least in part, for the mechanisms restricting their neurogenic potential.

The NFI factors have been shown to promote cell cycle exit of RPC^30^ and quiescence of Müller glia after injury^27^. Thus, we first assumed that Nfia expression should be downregulated in proliferating Müller glia. Contrary to our expectation, Nfia expression was maintained in rat Müller glia during injury-induced proliferation and significantly increased in mouse Müller glia after cell cycle reentry in dissociated cultures. Our findings agree with the recent report that Nfia is highly expressed in Müller glia-derived proliferating progenitors in the chick retina^31^. Nfia, while critical for maintaining differentiation and quiescence of Müller glia^27^, may act to drive proliferation once Müller glia are reprogrammed to a proliferation-competent state. It is also likely that Nfia may promote glial proliferation at young ages (this study) while inhibiting proliferation in older mice^27^. The possibility that Nfia may behave in an opposite manner to Nfib and Nfix in regulating proliferation cannot be also excluded. However, our data are not compatible with the pro-proliferative effects of Nfia, because Nfia levels were more highly correlated with S phase progression in P21 mature Müller glia, which were less proliferative than immature ones from the P10 retinas. The increased expression of Nfia in dividing cells may therefore be a homeostatic response aimed at blocking glial proliferation. This possibility is supported by the well-established anti-proliferative effects of NFI factors in the retina^27,30^. Alternatively, Nfia may promote glial cell fate and limit neurogenic potential of Müller glia as has been suggested previously in the chick retina^31^. Nfia, together with other NFI factors, plays a critical role in Müller glia specification during retinal development^30^. Also, Nfia has been implicated in astrocyte differentiation and induction of reactive astrogliosis after CNS injury^54–57^. In contrast to Pax6 and Vsx2, the association of Nfia levels with the cell cycle was stronger in mature Müller glia, possibly implicating this factor in a gliogenic nature of Müller glia proliferation in the mature mammalian retina.

Consistent with the previous reports^3–5,41^, injury induced cyclin D1 upregulation in Müller glia in both mice and rats, but the upregulation was more drastic in proliferating Müller glia in rats compared to non-proliferative mouse Müller glia, suggesting a relevance of cyclin D1 levels in species difference in proliferative potential of Müller glia^4,5^. The mechanism underlying the species difference in Müller glia activity remains unknown. Rat Müller glia may be intrinsically more proliferative, but the apparent difference in the MNU-induced ONL changes between the two species may indicate the relevance of the process of photoreceptor degeneration and/or photoreceptor/Müller glia interactions. When mouse Müller glia were forced to reenter the cell cycle by dissociated cultures, cyclin D1 levels were only transiently upregulated in G1 phase (cluster 2), followed by downregulation after S phase entry (cluster 3). These results are consistent with the previous reports that cyclin D1 levels increase in G1 phase and decline in S phase in asynchronously cycling cells^58,59^. The expression patterns of cyclin D1 were similar between P10 and P21 samples when examined at DIV2, in keeping with the results that the proportions of EdU+ and pH3+ cells were not significantly different between two ages. However, as regards P21 samples, G1-associated upregulation of cyclin D1 was no more evident at DIV5, reflecting the limited cell cycle reentry after DIV2. Cyclin D3 in mouse Müller glia was upregulated after injury in vivo, in agreement with the previous reports^3,4,15,42^. However, when Müller glia reentered the cell cycle by dissociation cultures, cyclin D3 levels were significantly downregulated after S phase entry, a change more noticeable in the P21 samples. This finding supports the previous report by Dyer and Cepko^43^ that cyclin D3 in mouse Müller glia is downregulated after injury-induced cell cycle reentry. Our findings suggest that injury stimulates cyclin D3 expression in non-proliferative Müller glia, but once they reenter the cell cycle, they rather downregulate this cyclin in S phase. Notably, rat Müller glia, most of which proliferate after injury, exhibited highly heterogeneous patterns of cyclin D3 expression in S phase; some cells increased cyclin D3 while others almost lost it, similar to the variability of D3 expression in dissociated mouse Müller glia. The significance of this heterogeneity in the Müller glia response remains unclear and further investigations are required to determine whether cyclin D3 plays any crucial role in cell cycle progression or other injury-induced responses of Müller glia.

Our quantitative image analysis is a simple but powerful method to delineate cell cycle-related protein expression in dissociated Müller glia. However, to define different cell cycle stages using cell-cycle markers is challenging and affected by selection of antibodies. We used MCM6 to label cycling cells, because it is well established as a pan-cell cycle marker in many tissues including the retina^5,46,60,61^ and specific antibodies raised from different species are available. MCM6 is a target of E2F transcription factor^62^ and upregulated in late G1 phase^49^. It is thus likely that MCM6 is not detected until late G1 phase of the first cell cycle and that cells in early G1 phase are contained in the cluster 1 (EdU−/MCM6−), which may account for the relatively small size of the cluster 2 (EdU−/MCM6+). Furthermore, MCM6 may be retained for some time after cell cycle exit^5,60^. Thus, MCM6+ cells in the cluster 2 (EdU−/MCM6+) and 3 (EdU+/MCM6+) may contain postmitotic cells or cells in the process of cell cycle exit, especially at DIV5. The inclusion of quiescent and proliferating populations in the same cluster may cause cell-to-cell variability within a cluster and underestimate the difference between clusters. The use of cell cycle markers allowing more stringent distinction between proliferation and quiescence may improve the quality of the present results.

In conclusion, we identified previously unknown age- and cell cycle-related expression patterns of RPC regulators in Müller glia. Proliferation is generally considered a hallmark of dedifferentiation, and proliferation of Müller glia is essential for retinal regeneration in zebrafish. However, proliferation of Müller glia in mammals is often gliogenic, rather than neurogenic, leading to glial scar formation. Thus, stimulating Müller glia proliferation alone may not be sufficient to enhance neurogenic potential of the mammalian retina. Understanding the mechanistic links between lineage-specific transcription factor expression and cell cycle progression would be essential to develop a strategy for stimulating regeneration of the mammalian retina.

## Methods

### Animals

C57BL/6J mice and Wistar rats were obtained from the Charles River Laboratories Japan (Yokohama, Japan). The animals were killed by decapitation or cervical dislocation under inhalation anesthesia with isoflurane. All animal experiments were conducted according to protocols approved by the institutional animal care committee of Tokyo Women’s Medical University and all methods were performed in accordance with the relevant guidelines and regulations of Tokyo Women’s Medical University. The study was reported in accordance with ARRIVE guidelines.

### Induction of retinal degeneration

Photoreceptor degeneration was induced by a single intraperitoneal injection of *N*-methyl-*N*-nitrosourea (MNU, Sigma-Aldrich, St. Louis, MO, USA) at the dose of 60 mg/kg body weight for mice (3 weeks old) and 70 mg/kg body weight for rats (5 weeks old), as reported previously^5^.

### Primary culture

The eyeballs were dissected and washed with Hank’s Balanced Salt Solution (HBSS (−), FUJIFILM Wako Chemicals, Osaka, Japan) containing 0.1% Gentamicin Sulfate (Nacalai Tesque, Kyoto, Japan). After incubating in HBSS(−)/Gentamicin at 37 °C for 1 h, the retinas were isolated, mechanically dissociated by pipetting, and centrifuged at 1000 rpm for 10 min. The pellet was resuspended in Dulbecco’s Modified Eagle Medium (DMEM) low glucose (Sigma-Aldrich, St. Louis, MO, USA) supplemented with 10% fetal bovine serum (FBS) and 1 µl/ml Penicillin–Streptomycin (PS), plated on polylysine-coated coverslips in 12-well dishes and cultured at 37 °C in a 5% CO_2_ incubator. To label mitotic cells, 5-ethynyl-2′-deoxyuridine (EdU, Thermo Fisher Scientific, Waltham, MA, USA) was added to the medium (8 μl/ml) after 1 day of culture.

### Immunofluorescence

The eyeballs were dissected and fixed in 4% paraformaldehyde in phosphate buffer (PB) for 1 h, rinsed in 15% and 30% sucrose in PB, and frozen with dry ice-isopentane. Cryostat sections were prepared at a thickness of 10 μm and stored at − 20 °C until use. For in vitro analyses, cultured cells were fixed in 4% paraformaldehyde in PB for 15 min and stored at 4 °C in PB. Samples were washed with phosphate-buffered saline containing 0.3% Triton X-100 (PBST), blocked with Blocking One or Blocking One P (Nacalai Tesque, Kyoto, Japan) for 30 min, incubated with primary antibodies in PBST at room temperature overnight, washed with PBST, and incubated with secondary antibodies in PBST for 30 min. Primary and secondary antibodies used are listed in Supplementary Table 1. The nuclei were counterstained with 4′,6-diamidino-2-phenylindole (DAPI). EdU labeling was performed using Click-iT Plus EdU Cell Proliferation Kit for Imaging, Alexa Fluor 647 (Thermo-Fisher-Scientific, Waltham, MA, USA) according to manufacturer’s instructions prior to primary antibody incubation. Fluorescence signals were examined by confocal laser scanning microscope (LSM710, Carl Zeiss, Jena, Germany).

### Image analysis

Müller glia cultures were subjected to quadruple staining for a Müller glia marker (Lhx2 or Sox9), MCM6, EdU, and a protein of interest. Confocal images were acquired with a 40× objective and analyzed using CellProfiler software 4.0.7 (http://www.cellprofiler.org). Müller glia nuclei were identified with Lhx2 or Sox9 labeling and fluorescence intensities (average and integrated) of MCM6, EdU, and target proteins within the nuclei were automatically measured. As confocal parameters were adjusted for each staining sample, intensities were statistically analyzed within a sample, but not compared between samples. Integrated intensities of EdU and target proteins in Müller glia grouped into four clusters were presented as scatter and box plots and analyzed statistically.

### Statistics

All statistical analyses were performed using IBM SPSS statistics software (ver. 19). Statistical comparisons were made using Student’s t-test or one-way analysis of variance (ANOVA) with Games–Howell post-hoc test. The Spearman correlation coefficient was used to assess correlations between EdU and target signal intensities. P < 0.05 was considered statistically significant.

## Supplementary Information

Supplementary Information.

## Data Availability

The datasets generated during and/or analyzed during the current study are available from the corresponding author on reasonable request.
