# Supplementary material for: Age- and cell cycle-related expression patterns of transcription factors and cell cycle regulators in Müller glia
Source: Sci Rep. 2022 Nov 15;12:19584. doi: 10.1038/s41598-022-23855-w (PMC9666513; doi:10.1038/s41598-022-23855-w)
Supplement: Supplementary file 1 — Supplementary Information. [file 41598_2022_23855_MOESM1_ESM.docx]

Supplementary Table S1. List of antibodies.

| **Antibodies** | **Dilution** | **Species** | **Source** |
| --- | --- | --- | --- |
| CyclinD1 | 1:200 | Rabbit | LabVision RM9104-S0 |
| CyclinD3 | 1:1000 | Mouse | Cell signaling 2936S |
| Glutamine synthetase | 1:5000 | Rabbit | Sigma G2781 |
| Lhx2 | 1:600 | Goat | Santa Cruz sc-19344 |
| MCM6 | 1:5000 | Goat | Santa Cruz sc-9843 |
| MCM6 | 1:2000 | Mouse | Santa Cruz sc-393618 |
| Nfia | 1:2000 | Rabbit | Atlas HPA006111 |
| Pax6 | 1:400 | Rabbit | Millipore AB2237 |
| Phospho-histone H3 | 1:5000 | Mouse | Upstate 05800 |
| Phospho-pRb | 1:2000 | Rabbit | Cell signaling 8516S |
| Sox9 | 1:2000 | Goat | RD Systems AF3075 |
| Sox9 | 1:2000 | Rabbit | Millipore AB5535 |
| Vsx2 | 1:2000 | Sheep | Exalpha X1179P |
| Donkey anti-Mouse IgG Alexa Fluor 405 | 1:1000 | Donkey | invitrogen A-48257 |
| Donkey anti-Mouse IgG Alexa Fluor 488 | 1:1000 | Donkey | invitrogen A-21202 |
| Donkey anti-Mouse IgG Alexa Fluor 555 | 1:1000 | Donkey | invitrogen A-31570 |
| Donkey anti-Rabbit IgG Alexa Fluor 488 | 1:1000 | Donkey | invitrogen A-21206 |
| Donkey anti-Rabbit IgG Alexa Fluor 555 | 1:1000 | Donkey | invitrogen A-31572 |
| Donkey anti-Goat IgG Alexa Fluor 488 | 1:1000 | Donkey | invitrogen A-11055 |
| Donkey anti-Goat IgG Alexa Fluor 555 | 1:1000 | Donkey | invitrogen A-21432 |
| Donkey anti-Sheep IgG Alexa Fluor 488 | 1:1000 | Donkey | invitrogen A-11015 |
| Donkey anti-Sheep IgG Alexa Fluor 555 | 1:1000 | Donkey | invitrogen A-21436 |
